# An Unusual Occurrence of *Nautilus macromphalus* in a Cenote in the Loyalty Islands (New Caledonia)

**DOI:** 10.1371/journal.pone.0113372

**Published:** 2014-12-03

**Authors:** Neil H. Landman, Royal H. Mapes, J. Kirk Cochran, Vincent Lignier, Daniel I. Hembree, Claire Goiran, Eric Folcher, Philippe Brunet

**Affiliations:** 1 Division of Paleontology (Invertebrates), American Museum of Natural History, New York, New York 10024, United States of America; 2 North Carolina Museum of Natural Sciences, Raleigh, North Carolina 27601, United States of America; 3 School of Marine and Atmospheric Sciences, Stony Brook University, Stony Brook, New York 11794, United States of America; 4 Laboratoire PPME (Pôle Pluridisciplinaire de la Matière et de l'Environnement), Université de la Nouvelle-Calédonie, BP R4, 98851 Nouméa cedex, New Caledonia; 5 Labex Corail & LIVE (Laboratoire insulaire du Vivant et de l'Environnement), Université de la Nouvelle-Calédonie, BP R4, 98851 Nouméa cedex, New Caledonia; 6 Institut de Recherche pour le Développement, BP A5, 98848 Nouméa cedex, New Caledonia; 7 Independent Researcher, 21 rue Louis Fablet, 94200 Ivry sur Seine, France; Universität Göttingen, Germany

## Abstract

Exploration of a landlocked cenote on Lifou (Loyalty Islands) revealed 37 shells of the cephalopod *Nautilus macromphalus* Sowerby, 1849, in saltwater on the cenote floor, approximately 40 m below the water surface. The occurrence of these shells is unusual because *N. macromphalus* is restricted to the open marine waters surrounding the island. All of the shells are mature, and nearly all of them are unbroken, with faded red-brown color stripes. We analyzed seven shells to determine their age. Radiocarbon dating yielded ages of 6380±30 to 7095±30 y BP. The ^238^U-series radionuclides ^210^Pb (half-life  = 22.3 y) and ^226^Ra (half-life  = 1600 y) also were measured. Two of the samples showed radioactive equilibrium between the nuclides, consistent with the old radiocarbon dates, but the other five samples showed excess ^210^Pb. When corrected for radioactive decay, the ^226^Ra activities were much greater than those found in living *Nautilus*. We conclude that exposure to high activities of ^222^Rn and ^226^Ra in the salty groundwater of the cenote altered the activities originally incorporated into the shells. Human placement of the shells in the cavity is rejected based on their radiocarbon age and the geometry of the cenote. The most probable explanation is that the animals entered the flooded karstic system through a connection on the seaward side at approximately 7,000 y BP, during an interval of slowly rising sea level. Unable to find an exit and/or due to anoxic bottom waters, the animals were trapped and died inside. The open connection with the sea persisted for ∼700 y, but after ∼6400 y BP, the connection was lost, probably due to a roof collapse. This is a rare example of *Nautilus* in a karstic coastal basin and provides a minimum age for the appearance of *N. macromphalus* in the Loyalty Islands.

## Introduction

**Figure 1:**
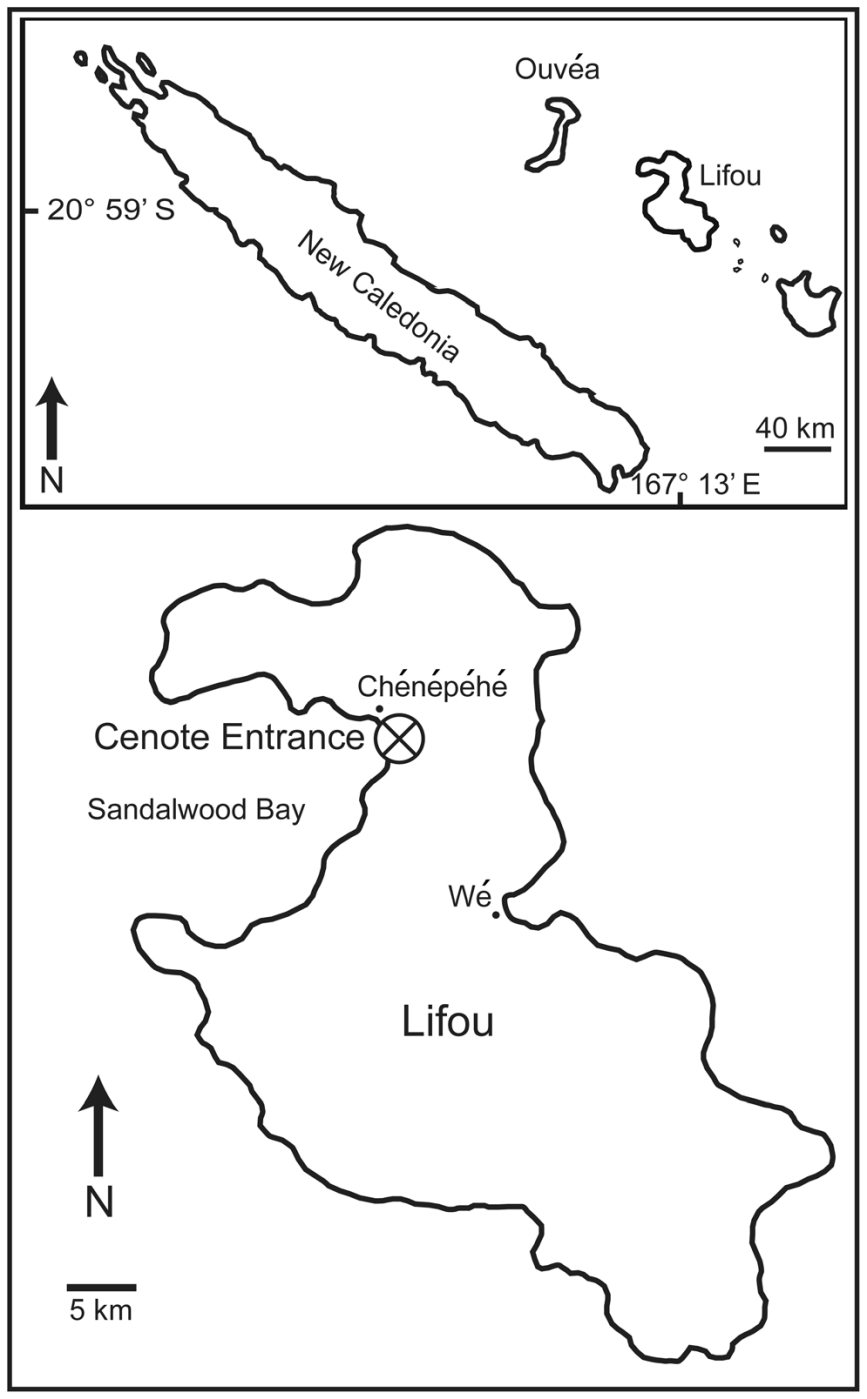
Location of New Caledonia and Lifou. Map showing location of Lifou within the Loyalty Islands, New Caledonia. The entrance of the Ani e Wee cenote on Lifou (marked with an X) is approximately 200 m from the coast.

Shells of *Nautilus macromphalus* Sowerby, 1849 [1], were discovered in a landlocked cenote near the west coast of Lifou in the Loyalty Islands (New Caledonia) during three diving expeditions between 2009 and 2011 (Fig. 1). No evidence of an open connection between the cenote and the sea is visible at this time. *Nautilus macromphalus* is endemic to these islands and usually occurs at depths between 50 and 500 m [2], [3]. Drifted shells of this species commonly wash up along the shore [4], but this is one of the few known occurrences of *Nautilus* shells in a coastal karstic basin. The abundance and relatively good preservation of the shells allow dating of the specimens to provide information on their origin and the time of appearance of *N. macromphalus* in the Loyalty Islands.

Lifou is the largest of the Loyalty Islands and is located 190 km northeast of New Caledonia. It is a raised atoll with coral limestones exposed along the margin and lagoonal biomicrities exposed at the center of the island. It has been uplifted approximately 100 m due to its position on the leading edge of the Australian plate prior to its subduction at the Vanuatu Trench [5], [6]. During the Pleistocene, an extensive karstic system developed on the island [7], especially along the coast, where caves periodically became flooded in response to glacioeustatic changes in sea level. The karstic features along the coast comprise anchialine (or anchihaline), habitats, which were defined by Holthuis [8] as “pools with no surface connection with the sea, containing salt or brackish water, which fluctuates with the tides”. This concept was later modified slightly to emphasize subterranean connections to the sea as well as terrestrial influences [9], [10].

## Methods

### Ethics statement

C. Goiran received administrative permission to collect *Nautilus* from the Province des îles Loyauté. She also obtained formal authorization from the Province Sud to export the empty *Nautilus* shells for scientific purposes. P. Brunet received permission to explore and collect the *Nautilus* in the cenote from the local chiefs who oversee the area in Lifou. The authors received funding for the dive in 2011 from the Eramet-Société Le Nickel, a mining company in New Caledonia, and Betco2, a marine transport company operating between Nouméa and the Loyalty Islands. This funding does not alter our adherence to PLOS ONE policies on sharing data and materials.

### Sample collection

The lake in the cenote can be reached without climbing equipment. Indeed, occasionally, local youth come to bathe there. Nevertheless, security requires the use of ropes to transport heavy equipment. Diving in the cave requires mastering the techniques of cave diving including diving in the dark under a ceiling with a life-line. The location of each shell was recorded with reference to a quadrant system (each square frame is 1 m×1 m). Shells were numbered, mapped, and photographed using the quadrant array. Six quadrants were established, and two to six shells were inventoried in each quadrant. The specimens were labeled to provide information on the sequence in which each shell was collected and the quadrant in which it was collected in, for example, N10QC indicates the tenth *Nautilus* shell collected, which was in the C quadrant. Locality information for sample ID1 was not recorded.

### ^210^Pb and ^226^Ra analysis

The shells were crushed into fine pieces, and ∼100 g of each shell were sealed in aluminum cans for analysis (leaving a few pieces over for future examination, reposited in the Division of Paleontology, American Museum of Natural History). After a delay of several weeks to ensure the attainment of equilibrium between ^226^Ra and ^222^Rn and daughters, the samples were counted on a 3800 mm^2^ Canberra planar intrinsic germanium gamma detector. Gamma emissions at 46 keV (^210^Pb) and 352 keV (^214^Bi) were used to calculate the activities of ^210^Pb and ^226^Ra, respectively. NIST SRM 4350B was used to determine the counting efficiency of ^226^Ra. Solution standards of varying density were prepared to calibrate the ^210^Pb measurements and correct for sample self-absorption of ^210^Pb gamma rays.

### ^14^C analysis

Following gamma spectrometry, several pieces from each shell were selected for radiocarbon analyses (the samples were destroyed in the process). Samples were converted to graphite, and ^14^C was measured via accelerator mass spectrometry at the WM Keck Carbon Cycle Accelerator Mass Spectrometry Laboratory (University of California, Irvine). Sample preparation backgrounds were subtracted, based on measurements of ^14^C-free calcite and normalization standards (OX-1) run with each set of samples. One sample also was rerun by selecting a large shell chip (>1 mm), crushing it coarsely, and then applying a 20–30% acid etch prior to ^14^C analysis.

### Setting

**Figure 2:**
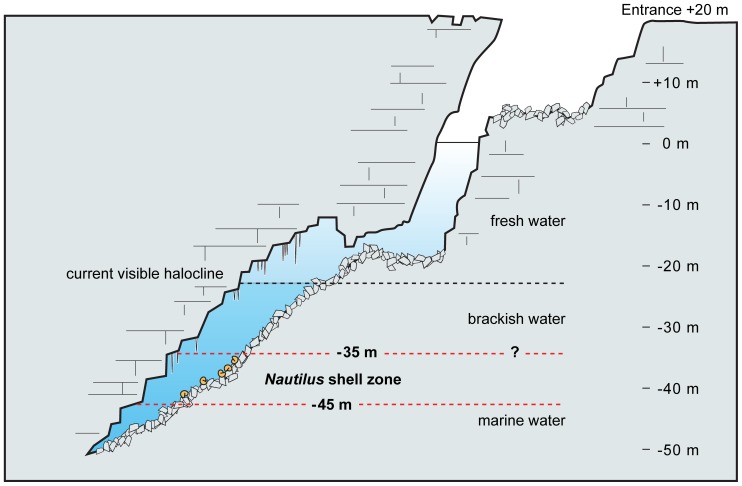
Vertical section of the Ani e Wee cenote. Schematic vertical section of the cenote (NNW-SSE) as determined by a dive in 2010. The entrance of the cenote is approximately 30 m in diameter. The *Nautilus* shells occur between 35 and 45 m. Because of the cenote geometry, a shell thrown into the opening by humans could not have come to rest in its present position.

The cenote is called “Ani e Wee” (also known as “Ani Angetre Ceu”) by the local indigenous people (Kanaks). It is located 200 m from the east coast of Sandalwood Bay (Baie de Sandal), south of the village of Chépénéhé (Fig. 1). Sandalwood Bay is a broad, shallow embayment and only exceeds a depth of 40 m at a distance of 8 km away from the coastline. The entrance of the cenote is a collapsed depression 30 m in diameter at the bottom of which is a lake 6 m wide, 20 m long, and 20 m deep (Fig. 2). The chasm opens up into a long gallery that forms a dogleg, and then dips down at an angle of 45° extending to a depth of 50 m. The roof of the gallery is covered with speleothems, and the floor is strewn with a scree slope of debris along the entire passageway. The blocks of debris are as much as 1 m in diameter.

The cenote contains a lens of fresh water on the surface, brackish water below, and brine at depth (Fig. 2). Closest to the entrance area, the sediments are dark, viscous, and mainly consist of organic matter and detrital carbonate grains. Between depths of 25 m and 35 m, the sediment is light brown and consists of organic matter and carbonate silt. Below 35 m, sediment is sparse. It is light tan, silty to clayey carbonate mud only a few millimeters thick. The three sediment types correspond respectively to fresh water to a depth of ∼25 m (dark organic sediments), brackish water between ∼25 and 35 m (light brown sediments), and marine water below ∼35 m (light tan sediments). The water level shows some variation with the tides, suggesting a subterranean connection with the sea.

## Results

### Description of Nautilus shells

**Figure 3:**
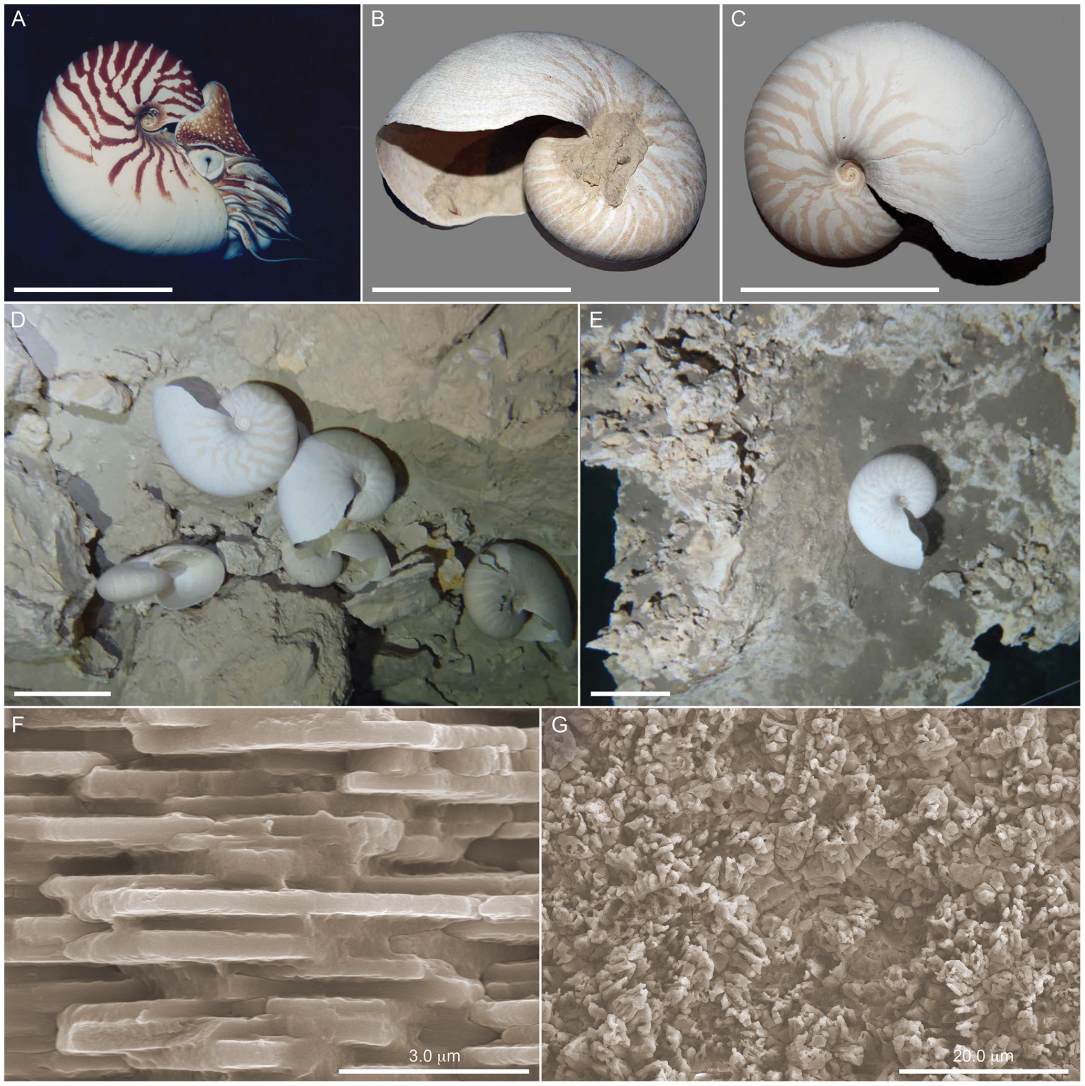
Specimens of *Nautilus macromphalus*. A. Live specimen of *Nautilus macromphalus* from New Caledonia. B, C. Specimens of *N. macromphalus* from the Ani e Wee cenote with faded red-brown color stripes. D, E. Still frames from a video clip showing specimens of *N. macromphalus* in situ on the rocky cenote floor. F, G. Scanning electron micrographs of the cross-section and surface, respectively, of the shell wall of a specimen of *N. macromphalus* from the Ani e Wee cenote.

Altogether, a total of 37 shells of *Nautilus macromphalus* were observed on the cenote floor between 35 and 45 m below the surface of the water. The shells are oriented randomly with most shells lying on their sides in and among the fallen blocks (Fig. 3D, E). Nearly all of the shells are intact, but at least one of them is damaged, probably from a fallen block. A few millimeters to a few centimeters of sediment partially cover the shells, filling the umbilical opening and body chamber. Some of the shells are cemented to the floor. No living organisms or remains of other marine invertebrates (or vertebrates) are associated with the shells.

Seven shells were collected for further analysis. All are mature individuals as indicated by their size (15–16 cm in diameter) and color pattern, with the red-brown color stripes disappearing on the ventral area of the body chamber (Fig. 3B–E). The shells do not exhibit any borings, repaired shell injuries, or evidence of epizoa. No periostracum is present, and the red-brown color stripes are faded, so that the surface of the shell presents a chalky appearance (compare Fig. 3A to 3B, C). In close-up, the shell surface is granular (Fig. 3G), reflecting dissolution of the outer prismatic layer. However, no mineral deposits are present on the surface. In cross-section, the nacreous microstructure of the shell is degraded. The nacreous tablets are slightly fused together, giving the impression of minor dissolution and reprecipitation (Fig. 3F). According to the preservation index of nacre established by Cochran et al. [11], the PI is 3.5 (good to very good).

### Dating

**Figure 4:**
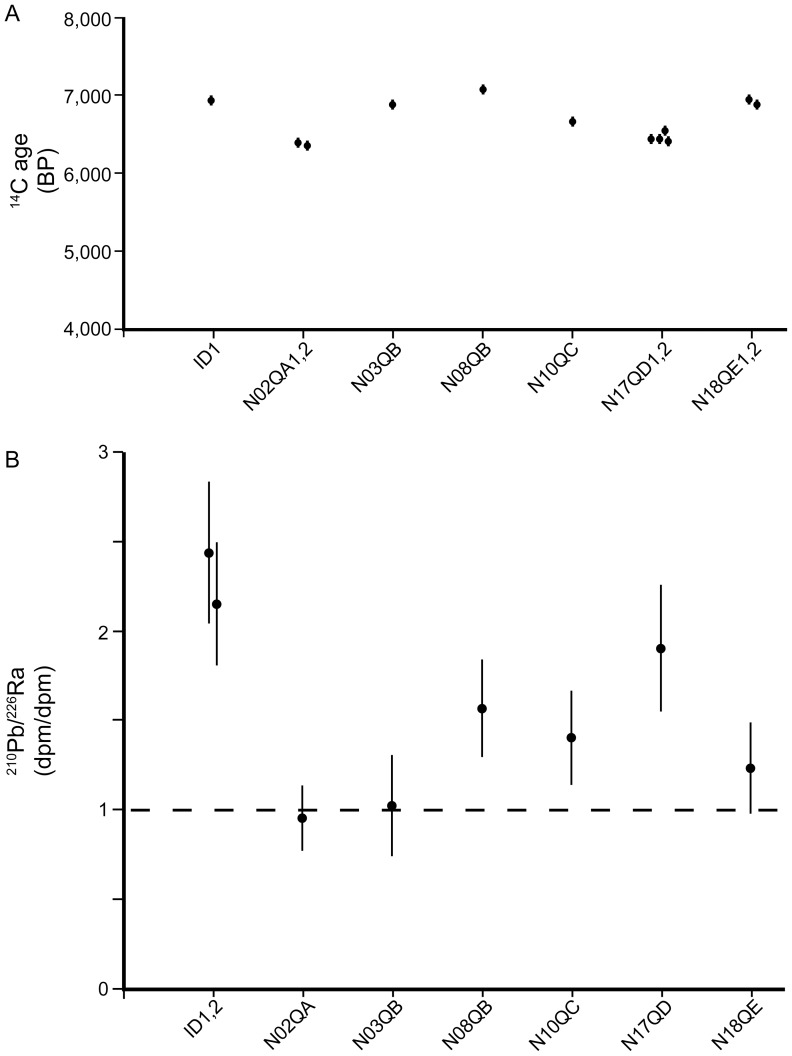
Radiocarbon and ^210^Pb/^226^Ra data for *N. macromphalus* from the Ani e Wee cenote. A. Radiocarbon dates range from 6380±30 to 7095±30 y BP. B. Activity ratio of ^210^Pb/^226^Ra. Two of the specimens show an activity ratio of approximately 1.0 suggesting an age of >100 years, which is consistent with the old radiocarbon ages. The other specimens show an excess of ^210^Pb. Correcting the ^226^Ra activities for decay (∼7000 years) produces values much greater than those observed in living *Nautilus*. Shells were likely exposed to high activities of ^222^Rn and ^226^Ra in the groundwater of the cenote such that ^210^Pb and ^226^Ra were adsorbed on the shell surfaces elevating their activities.

The shells were ground up into small pieces for radiocarbon dating. The radiocarbon dates average 6764±30 y BP with a range from 6380±30 to 7095±30 y BP (Fig. 4A; Table 1). Two of the shells each were analyzed twice, and one of the shells was analyzed three times, all with nearly identical results. In addition, one of the samples was re-analyzed after treatment with mild acid to remove any contaminants on the surface and yielded nearly the same result (Fig. 4A). The ^238^U series radionuclides ^210^Pb (half-life  = 22.3 y) and ^226^Ra (half-life  = 1600 y) were also measured (Fig. 4B; Table 2). One of the samples was measured twice with nearly identical results. Two of the samples show radioactive equilibrium between the nuclides, consistent with the old radiocarbon dates. However, the other five samples show excess ^210^Pb.

**Table 1:** Radiocarbon data for specimens of *Nautilus macromphalus* from the Ani e Wee cenote on Lifou, Loyalty Islands, New Caledonia.

Specimen ID	Fraction modern*	±	D^14^C (‰)*	±	^14^C age (y BP)*	±
1D	0.4222	0.4222	−577.8	1.5	6925	30
N02QA	0.4509	0.4509	−549.1	1.5	6400	30
"	0.4519	0.4519	−548.1	1.5	6380	30
N03QB	0.4238	0.4238	−576.2	1.5	6895	30
N08QB	0.4134	0.4134	−586.6	1.5	7095	30
N10QC	0.4357	0.4357	−564.3	1.5	6675	30
N17QD	0.4482	0.4482	−551.8	1.5	6445	30
"	0.4499	00.4476	−5552.4	1.5	6455	30
"	0.4476	0.4499	−550.1	1.5	6415	30
"**	0.4483	0.0013	−551.7	1.3	6445	25
N18QE	0.4208	0.4208	−579.2	1.5	6955	30
"	0.4240	0.4240	−576.0	1.5	6895	30

*Radiocarbon concentrations are expressed as fractions of the modern standard, D ^14^C, and conventional radiocarbon age, following the usage in Stuiver and Polach [25].

**Replicate sample was acid-cleaned before analysis.

" Denotes replicate radiocarbon measurement on indicated sample.

**Table 2:** Activities of ^210^Pb and ^226^Ra in specimens of *Nautilus macromphalus* from the Ani e Wee cenote on Lifou, Loyalty Islands, New Caledonia.

Specimen ID	^210^Pb (dpm/g)	±	^226^Ra (dpm/g)*	±	^210^Pb/^226^Ra (dpm/dpm)	±	Excess ^210^Pb (dpm/g)	±	Age (y)**
1D1	0.310	0.045	0.127	0.008	2.44	0.39	0.183	0.045	<100 y
"	0.317	0.046	0.147	0.010	2.15	0.34	0.169	0.047	<100 y
N02QA	0.174	0.030	0.181	0.012	0.96	0.18	−0.007	0.032	>100 y
N03QB	0.167	0.045	0.162	0.007	1.03	0.28	0.005	0.045	>100 y
N08QB	0.241	0.039	0.154	0.009	1.56	0.27	0.087	0.040	<100 y
N10QC	0.215	0.037	0.153	0.009	1.40	0.26	0.062	0.038	<100 y
N17QD	0.211	0.036	0.111	0.008	1.90	0.35	0.100	0.037	<100 y
N18QE	0.136	0.026	0.109	0.006	1.24	0.25	0.026	0.027	≤100 y

*^226^Ra activity at time of shell collection.

**Approximate age based on presence or absence of excess ^210^Pb in sample.

## Discussion

The alteration observed in the nacreous microstructure of the *Nautilus* shells could affect the determination of the radiocarbon age. Mild acid leaching of the surface of one shell sample and re-analysis for radiocarbon yielded no difference in age, suggesting that any alteration is integral to the shell itself and not caused by the precipitation of a surface phase. Indeed, Cochran et al. [11] demonstrated that as preservation declines in fossil ammonoids and nautiloids, the nacreous tablets begin to fuse together, probably due to the precipitation of SrCO_3_. The added carbonate may be derived from dissolution and re-precipitation of the shell itself or from the dissolved inorganic carbon (DIC) reservoir in the water surrounding the shells. In the former case, the radiocarbon age would be unaffected, but, in the latter, carbon of a different age would be added to the shell. Because the shell preservation of the Lifou samples is assessed as good-to-very good (PI = 3.5), the added carbonate likely accounts for less than ∼5% of the shell carbon. If the DIC were imprinted with pre-bomb C (fraction modern  = 1), then a 5% addition would have produced a radiocarbon age ∼500 y younger than the true age of the shell. In contrast, if the DIC in the cenote is old (e.g. 10,000 y, derived from the carbonate rocks of the island), the alteration would have produced a shell radiocarbon age ∼150 y older than the true age. We therefore conclude that the radiocarbon ages of the shells are within several hundred years of their true ages.

*Nautilus* secretes its shell with excess ^210^Pb (half-life  = 22.3 y; ^210^Pb/^226^Ra >1.0) and with ^226^Ra activities of 0.04–0.15 dpm/g [12], [13]. The two shell samples with a ^210^Pb/^226^Ra activity ratio of approximately 1.0 suggest an age of >5 half-lives of ^210^Pb or ≥100 years (Table 2), which is consistent with the old radiocarbon dates of these specimens (Table 1). However, in the other five specimens, the ratio is greater than 1, suggesting a younger age. Correcting the ^226^Ra (half-life  = 1600 y) activities measured in the samples for decay over ∼7000 y produces values much greater than those observed in present-day *Nautilus* [12], [13]. The saline groundwaters of a cenote in the Yucatán, Mexico, have very high dissolved ^226^Ra activities [14], and a similar situation probably exists in the cenote in Lifou. Activities of dissolved ^222^Rn also would be elevated in such a system, and decay of ^222^Rn would produce ^210^Pb that could be adsorbed onto the surfaces of the shells or be incorporated into the shell wall during alteration. These processes probably altered the ^210^Pb and ^226^Ra activities in the shells, and suggest that the use of ^210^Pb/^226^Ra disequilibrium is not an appropriate method for dating these specimens.

We conclude that the average age of the shells of *Nautilus macromphalus* in the cenote is ∼6800 y BP. The question remains of how the shells arrived in the basin. Human placement of the shells in the gallery is rejected for two reasons. The first is the geometry of the cenote (Fig. 2). The cenote entrance opens up to a freshwater lake. Any shell thrown in by hand would have landed at the bottom of the pool at a depth of 20 m and not along the angled passageway where the shells occur. In addition, the first settlers on Lifou probably arrived at approximately 2500 y BP based on radiocarbon dating of hand stencils in nearby caves [15]. Thus, the average radiocarbon age of 6800 y BP predates the arrival of humans.

Today, the circulation of marine waters in the cenote is limited, as indicated by the reduced tidal rise and fall of the water level. However, the ages of the shells indicate that the passageway from the ocean to the cenote was open and accessible to *Nautilus*. The average date of 6800 y BP approximately coincides with an interval of slowly rising sea level, following the last rapid rise due to glacioeustatic processes (Fig. 9 in [16]). As a result, the ocean must have flooded the karstic system, perhaps multiple times. The absence of other organisms in the cenote suggests that they either did not enter the passageway, or if they did, they died inside and were dissolved away. The fact that more ancient and younger *Nautilus* shells have not been recovered suggests that the passageway to the cenote was open for a restricted length of time and then was blocked. This blockage was probably caused by a roof collapse, as indicated by the scree slope of limestone blocks. Similar collapses have been reported in coastal karstic systems elsewhere [17]. We conclude that *Nautilus* were able to enter what is now the cenote for a span of approximately 700 years.

The specimens may have arrived of their own volition or by post-mortem drift. The excellent preservation of the shells without broken edges or encrusting epizoans suggests that the animals swam rather than floated in. Nevertheless, it is surprising that the *Nautilus* ventured so far into shallow water so close to shore. However, in New Caledonia, *Nautilus* have occasionally been observed in shallow water during the austral winter months [2], [3], [18]. Willey [19] noted that “…in Sandal Bay [Sandalwood Bay], Lifou, in the Loyalty Group…*Nautilus* migrates at night from deep water into as little as three fathoms.” R. Davis (pers. comm., as cited in [18]) also reported catching a specimen by hand-line at night in Sandal Bay at an apparent depth of less than 5 m. Most of the specimens observed in shallow water are mature [2], which matches the description of the shells in the cenote. The animals may have been searching for benthic crustaceans such as hermit crabs and lobsters, which have been reported in the crop contents of *Nautilus* caught between 10 and 30 m [20]. They may also have been attracted by an unusual food item, for example, an animal corpse that fell from the land into the abyss, which they detected by smell. Alternatively, the *Nautilus* may have taken refuge in the crevices and caves, trying to avoid predators such as turtles, triggerfish, and sharks.

Once in the cavity, the animals might not have been able to find the exit and died of starvation. Alternatively, they may have died due to suffocation from anoxic bottom waters, which have been reported in cenotes on Ouvéa in the Loyalty Islands [21]. Decomposition of the soft body may have caused the shells to float upward, a common phenomenon after death [22]. However, the shells probably did not reach the surface of the freshwater pool because of the cenote geometry. Eventually, such shells would have become waterlogged and sunk to the floor where they accumulated at a depth of 35 to 45 m.

Shells of *Nautilus macromphalus* also have been reported in the Manev cenote on the east coast of Lifou, near the village of Wé (pers. comm., S. Pujol). They are more completely buried in the sediment and less well preserved than those in the present study. In both the Manev and the Ani e Wee cenotes, the cavities are close to the shore, and must have been flooded by sea level rise during the Holocene.

Although *Nautilus* is considered a “living fossil,” the actual fossil record of this genus is very poor, making the study of its evolutionary history and migratory pathways difficult. The only record of a fossil *Nautilus* is a specimen attributed to *N. pompilius* from the Philippines, which, based on microfossils in the surrounding rock matrix, appears to be early Pleistocene [23]. The maximum age of the specimens in the cenote on Lifou provides a minimum age of the appearance of *N*. *macromphalus* in the Loyalty Islands. According to phylogenetic reconstructions based on molecular data, *N*. *macromphalus* diverged from the common ancestor of the *Nautilus* populations in Fiji, Vanuatu, and American Samoa [24]. Our radiocarbon data reveal that this event must have occurred no later than 7100 y BP.

## Conclusions

The occurrence of *Nautilus macromphalus* in a cenote on Lifou in the Loyalty Islands is unusual because this species is restricted to the open marine waters surrounding the islands. Human placement of the shells in the cenote is rejected based on their radiocarbon age and the geometry of the cenote. The well-preserved state of the shells suggests that the specimens entered as live animals from Sandalwood Bay. The radiocarbon dates indicate that this occurred following the last rapid rise of sea level. Once inside the cenote, the animals became trapped and died of starvation or suffocation. The passageway may have persisted for approximately 700 y after which a roof collapse probably blocked the seaward entrance. The radiocarbon analysis indicates that *N*. *macromphalus* was present in the Loyalty Islands at least as long ago as 7100 y BP.
