# Correction: An Unusual Occurrence of *Nautilus macromphalus* in a Cenote in the Loyalty Islands (New Caledonia)

**DOI:** 10.1371/journal.pone.0118532

**Published:** 2015-03-05

**Authors:** The PLOS ONE Staff

The affiliation for the fifth author is missing. Daniel I. Hembree is not affiliated with #2. He is affiliated with: Department of Geological Sciences, Ohio University, Athens, Ohio 45701, United States of America.
